# Durable graphene-based alkyd nanocomposites for surface coating applications

**DOI:** 10.1007/s11356-024-33339-1

**Published:** 2024-05-03

**Authors:** Mohamed S. Selim, Abdelaziz N. El-hoshoudy, ElSayed G. Zaki, Ashraf M. EL-Saeed, Ahmed A. Farag

**Affiliations:** Egyptian Petroleum Research Institute (EPRI), Nasr City, Cairo 11727 Egypt

**Keywords:** Sustainability, Vegetable oils, Alkyd/graphene nanocomposites, Protective coatings, Surface durability, Anti-corrosion properties

## Abstract

Recently, the scientific community’s main goal is the long-term sustainability. Vegetable oils are easily accessible, non-depletable, and cost-effective materials. Vegetable oils are used to prepare polymeric alkyd surfaces. Novel and exciting designs of alkyd/graphene nanocomposites have provided eco-friendly thermal stability and protective coating surfaces. This review has briefly described important graphene-based alkyd nanocomposites along with their applications as protective coatings. These alkyd composites have high hydrophobicity, corrosion resistance, and durability. Graphene-based alkyd nanocoatings have many industrial and research interests because of their exceptional thermal and chemical properties. This work introduces an advanced horizon for developing protective nanocomposite coatings. The anti-corrosion properties and coatings’ longevity may be improved by combining the synergistic effects of hybrid nanofillers introduced in this work.

## Introduction

Designing corrosion-protection coatings generated from vegetable oil represents a worldwide purpose for environmental sustainability [Bhat et al. 2024; Li et al. 2023]. Numerous environmentally friendly or “green” technologies have arisen from sustainable sources including plant oils (particularly vegetable oils) [Ebrahimnezhad-Khaljiri and Ghadi 2024]. Corrosion costs billions of dollars annually and has negative economic impacts for metal surfaces [Teijido et al. 2022]. Some of these negative consequences may be lessened with the use of protective surface coatings [Maruthi et al. 2024]. Vegetable oils’ chemical transformations are a broad array of renewable resources that offer a variety of surface functions. Vegetable oils are mostly composed of triglycerides (93–98%); the remaining fraction is made up of diglycerides, monoglycerides, and phosphoglycerides. Vegetable oils have been extensively employed for eco-friendly coating applications [Yin et al. 2024; Bhat et al. 2024].

Epoxies, acrylics, vinyl, polyesters, and polyurethane are examples of petroleum-based materials that are frequently expensive, hazardous, non-biodegradable, and produce high levels of volatile organic compounds (VOCs) when used in paint applications. Vegetable oils have fluidity characteristics, which provide cost-effectiveness, non-toxicity, biodegradability, and the requirement for no or low solvents. Vegetable oil coatings can be used in the packaging of papers, self-healing coatings, electrical insulation, and biodegradable anti-microbial, anti-fouling, and corrosion-resistance applications. Although they have extensive aliphatic hydrophobic chains, they are usually lacking in toughness, water solubility, and mechanical durability [Andrew and Dhakal 2022]. Vegetable oil-based coatings showed improved tensile strength, hardness, and surface robustness when compared to their petroleum-derived counterparts. Vegetable oil chains are flexible because they contain long aliphatic fatty acid chains. The final coated film is created by combining polymeric matrixes with derivatives of vegetable oils. These derivatives work as reactive diluents and combine the polymer and vegetable oils [Luleburgaz et al. 2024]. The goal of the study is to produce vegetable oil–based coatings with low- or zero VOCs. The free mobility of the polymer might be hampered during the polymerization processes by increased viscosity.

## Coating properties of alkyd resins

Alkyds are polyesters modified with oils’ unsaturated fatty acids through a polycondensation reaction. Alkyds have significant applicability as binders in surface coatings. The alkyd resins are categorized into (1) short alkyds with 30–42%, medium alkyds with 43–54%, long alkyds with 55–68%, and very long alkyds with > 68%, based on the percentage weight fraction of vegetable oils [Dizman and Kaçakgil 2022]. The two main methods for producing alkyds are the monoglyceride and fatty acid processes. There are two types of alkyds: drying and non-drying alkyd resins. The drying alkyds contain polyunsaturated fatty acids and drying vegetable oils. While the non-drying alkyd resins are made from fatty acids of non-drying vegetable oils. Alkyds exhibit high durability and gloss retention while suffering from poor resistance against chemicals (especially alkaline) because of the presence of many ester units [Chardon et al. 2021]. Since fatty acids are made from sustainable resources, their lower cost as alkyd components also makes them more affordable [Villada et al. 2023]. Alkyds were traditionally dried in air or at a higher temperature using an oven. The drying of alkyd coatings is attributable to the auto-oxidation process of the reactive units. Common driers such as lead carboxylates could be replaced by calcium, zinc, zirconium, and cobalt to speed up the drying process [Erich et al. 2017; Chardon et al. 2021].

Alkyd-based chlorinated rubber seed oil had greater drying properties and flame retardancy compared to their unchlorinated equivalents [Otabor et al. 2019]. Alkyd paints treated with linseed oil and sunflower oil were applied for marine and industrial environments [Heiskanen et al. 2010]. Electrochemical impedance spectroscopy and polarization curves were used to investigate their corrosion-related characteristics [Hoshi et al. 2024; Mehrian et al. 2024]. Reactive diluents are used in the formulation of low solvent-content of alkyd paints. Nuclear magnetic resonance and mass spectrometry were used to examine the crosslinking processes of high-solid alkyd paints when reactive diluents were presented [Flores et al. 2019; da Filicaia et al. 2023].

Research on novel alkyd coating systems has been actively pursued during the past 10 years. Alkyd nanocomposite coatings, waterborne coatings, and organic/inorganic coatings can introduce better surface properties than their conventional equivalents [Chek and Ang 2024; Abd El-Ghaffar et al. 2024]. Nano Fe_2_O_3_/alkyd waterborne coatings developed by Dhoke and Khanna have demonstrated outstanding resistance against abrasion, scratch, and corrosion [Dhoke and Khanna 2009]. Waterborne alkyd surfaces filled with nano-ZnO exhibited strong mechanical, anti-corrosion, and ultraviolet (UV) resistance. In silicone-modified alkyd waterborne coatings, the addition of ZnO nanoparticles enhanced the coatings’ heat resistance and mechanical characteristics [Jiao et al. 2021; Pathan and Ahmad 2013a, 2013b].

## Hyperbranched alkyd nanocomposites

Because of the hyperbranched polymers’ distinct characteristics and greater availability compared to dendritic macromolecules, they have attracted great attention [Belgaonkar and Kandasubramanian 2021; Selim et al. 2019]. Hyperbranched polymer manufacturing is more practical and affordable when compared to dendrimer production [Thalji et al. 2021]. There have recently been new artificial approaches established using highly branched alkyd resins and controlled molecular weight dispensations [Chardon et al. 2021]. The applications of hyperbranched polymers may be constrained by their relatively high non-uniformity and undefined arrangement. These macromolecules are polydisperse more than dendrimers because of their random distribution of branching points throughout the structure preparation. Coating resins with hyperbranched moieties represent the best ways to reduce VOCs. These polymers were reported as compact three-dimensional structures with low viscosity, even at high molecular weights. Therefore, the development of hyperbranched-based nanocomposites may reduce the use of VOC solvents while enhancing surface properties. Recently, nanosilver was embedded in hyperbranched urethane alkyd using an in situ method. The coatings were non-leaching and had a high level of bacterial resistance to *Serratia marcescens* bacteria. This resin has higher mechanical properties and requires less solvent in the coating’s composition.

Well-distributed carbon-based fillers were produced as a result of interactions between delocalized electrons of surfactants and the carbon nanofiller’s electron clouds as well and the hydrogen bonding between the alkyd backbone and the surfactant. These interactions could decrease the van der Waals forces between the carbon fillers and the resin. Due to the multiwall carbon nanotube (MWCNT) and graphene’s greater aspect ratio, surface area, and interfacial interaction with the alkyd backbone’s filler, they outperformed carbon black in terms of matrix dispersion. Alkyd resins filled with graphene and MWCNTs demonstrated improved cohesiveness, scratching, and elasticity [Bhattacharya 2016]. Alkyd/graphene nanocomposite showed higher corrosion protection than the unmodified alkyd due to nanofillers’ superior efficiency in inhibiting the oxidation and hydration of oxygen and water. Thus, alkyd/graphene nanocomposite exhibited high mechanical and anti-corrosion performance [Naik and Ratna 2015]. Additionally, the sheet structure helped it withstand corrosion. Drying times are shortened by silicone modification, which also increases coating-substrate adhesion and hardness [Du et al. 2021]. Interest in creating innovative nanostructured materials with minimal VOC emissions has increased. Vegetable oil-based alkyds have excellent durability and corrosion protection [Thakur and Karak 2013]. Inorganic nanofillers can enhance the performance of alkyd resin [Selim et al. 2015; Selim et al. 2017a]. The diameter size, shape, and loading percentage of inorganic nanofillers can all have a significant impact on the characteristics of polymer resin [Askar et al. 2021; Selim et al. 2017b; Selim et al. 2020a, 2020b].

**Fig. 1:**
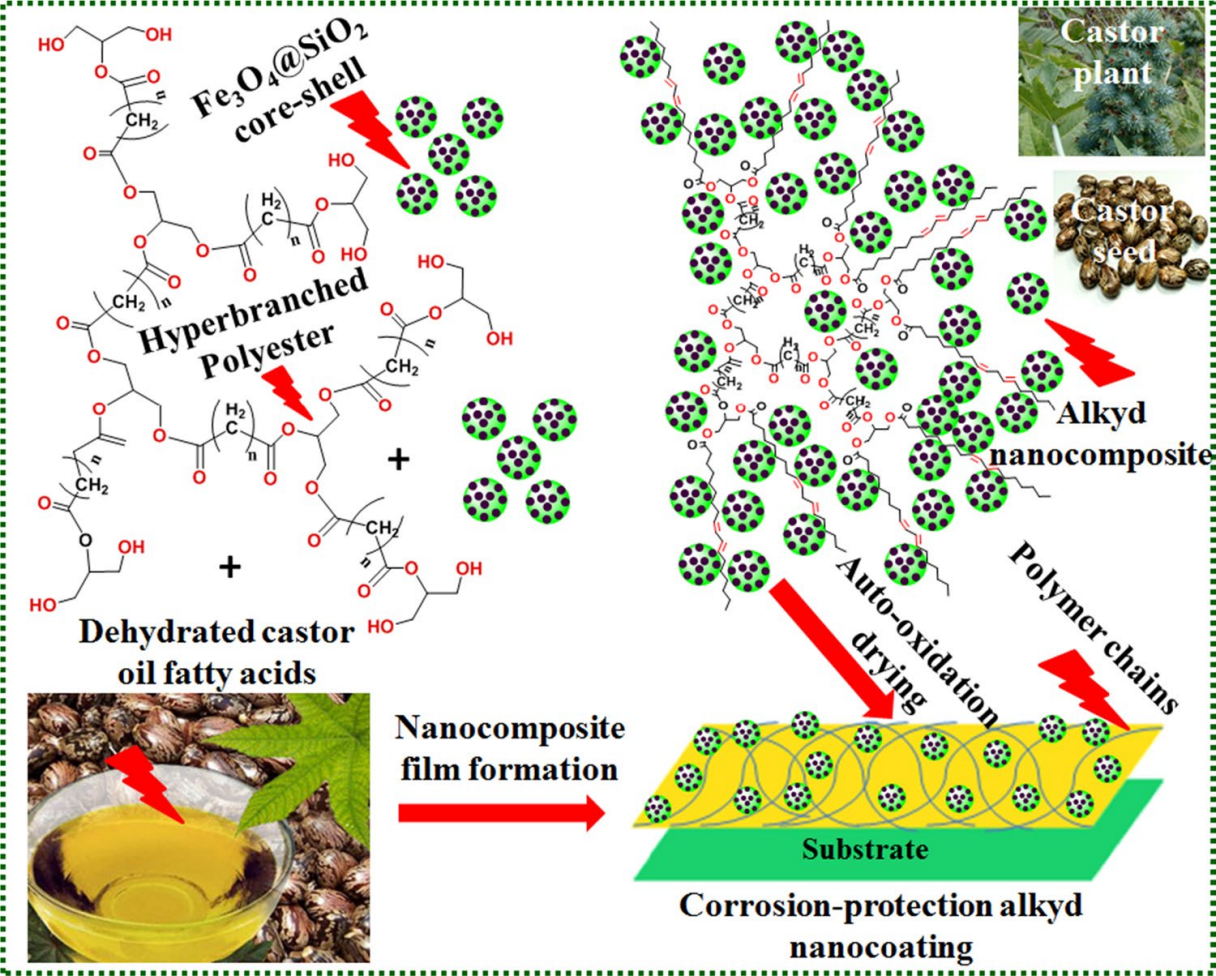
Ex situ approach for the synthesis of dehydrated castor oil-based alkyd filled with Fe_3_O_4_@SiO_2_ nanofillers as nanocomposite coatings. The used driers are calcium, cobalt, and zirconium octoates; the alkyd films were dried through auto-oxidation curing (Selim et al. 2017a). Copyright 2017; reprinted with Elsevier’s permission

Bifunctional nanostructured materials have drawn a lot of interest due to their dual structure and exceptional characteristics [Mechili et al. 2022; Kumar et al. 2018; Selim et al. 2022]. Active sites are provided by large surface area magnetite nanospheres for a range of applications [Sirelkhatim et al. 2015]. The breakdown of nanoparticles is prevented while simultaneously enhancing their biocompatibility by coating their surfaces with Stöber SiO_2_ core-shelled with Fe_3_O_4_ NPs [Wu et al. 2023]. The Fe_3_O_4_@SiO_2_ hybrid nanofillers combined the chemical composition and crystallinity of the two materials [Zhang et al. 2016; Hui et al. 2011]. Hyperbranched alkyd resin filled with Fe_3_O_4_@SiO_2_ NPs was employed as durable surface and coating material (Fig. 1) [Selim et al. 2017a]. Well-distributed core-shell of Fe_3_O_4_@SiO_2_ spheres were produced without aggregate. The produced coating film displayed exceptional stability in a salt fog environment, surface adhesion, and corrosion resistance by bifunctional nano-Fe_3_O_4_@SiO_2_ particles.

**Fig. 2:**
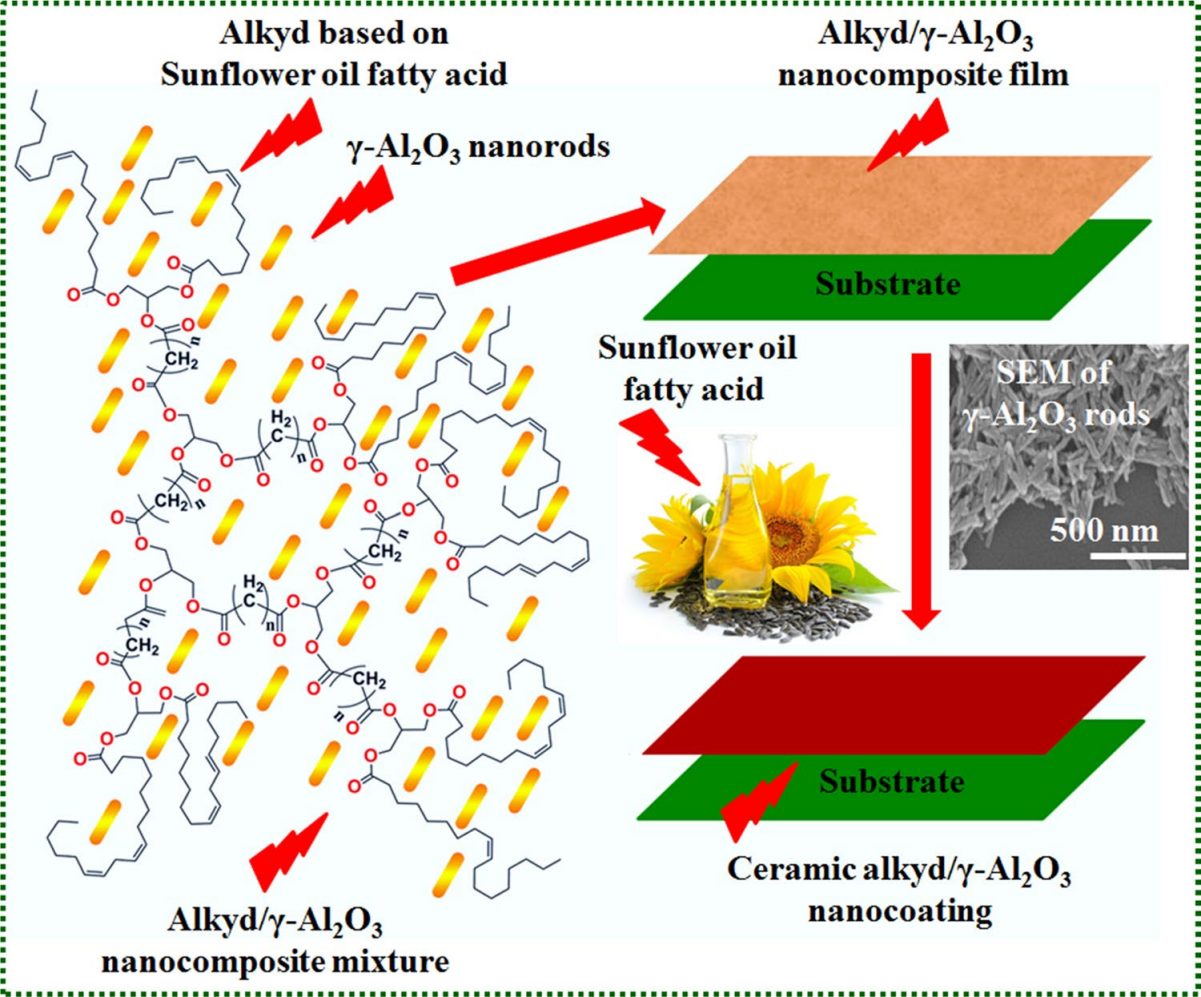
Alkyd produced from sunflower oil that contains nanorods of γ-Al_2_O_3_ and is filled with this material (Selim et al. 2018a, 2018b). Copyright 2018; reprinted with Elsevier’s permission

Eco-friendly hyperbranched alkyd resin enriched with γ-Al_2_O_3_ nanocomposites was prepared via the solution casting method and cured using an auto-oxidation process (Fig. 2) [Selim et al. 2018a]. The effects of loading different concentrations of ceramic nano γ-Al_2_O_3_ rods in the polymeric alkyd resins are that the coated nanocomposite films were remarkably resistive against the salt spray environment. They also showed high mechanical durability, water-repellency, and resistance against heat for the well-dispersed alkyd/γ-Al_2_O_3_ (0.5 wt.) nanocoatings.

**Fig. 3:**
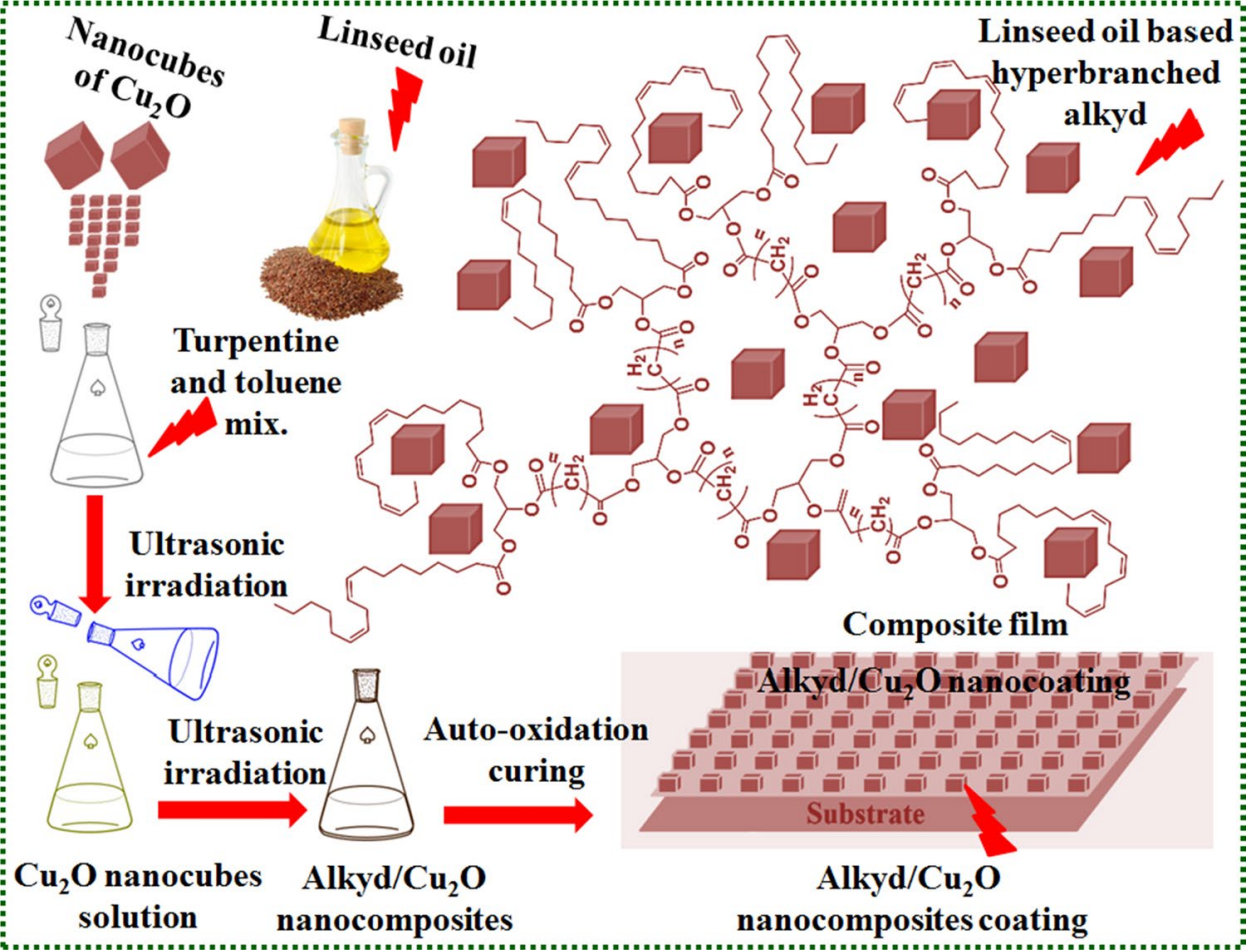
Hyperbranched alkyd (based on linssed oil) enriched with Cu_2_O nanocubes for applying as a protective nanocomposites coating (Selim et al. 2018b). Copyright 2018; reprinted with consent from RSC

Environmentally friendly linseed oil-based alkyd/Cu_2_O nanocomposites were prepared using a solution-casting procedure (Fig. 3) [Selim et al. 2018b]. A simple A_2_ + B_3_ approach was used to create a polyester with a hyperbranched moiety using natural and functional monomers. The developed well-dispersed alkyd/Cu_2_O nanocomposites showed outstanding physicomechanical and anti-corrosion properties.

Wang et al. (2012) reported the creation of elastomers based on soybean oil that had outstanding processability and changeable properties. One of the most effective methods for reducing VOCs in alkyd coatings is to create polymers with hyperbranched structures. Even at high molecular weight, these polymers display low viscosity due to the absence of constrictive interchain entanglements. The necessity for numerous purification processes can be avoided by producing hyperbranched alkyds in a single step [Jovičić et al. 2020].

Hyperbranched alkyd matrixes are produced through polyesterification reactions between the fatty acids in vegetable oils and polyesters. They exhibited outstanding flexibility, substrate-coating adhesion, and abrasion resistance [Duarte et al. 2024]. Hyperbranched alkyd polymers with higher solid contents, solubilities, and lower melting viscosities than their linear analogs were developed [Sair et al. 2023]. However, they have substantial defects, such as poor mechanical properties and weak alkaline resistance. Hyperbranched polyurethanes offer exceptional mechanical and physical characteristics; however, they have a high resin viscosity problem. Zhang et al. (2012a, 2012b) studied the characteristics of the polyurethane coating produced by the interaction of isocyanate trimers with hydroxylated polyester. Thakur and Karak (2013) reported developing castor oil-derived polyurethane with hyperbranched moiety and better coating properties. Hyperbranched alkyd/polyurethane copolymer can lead to the creation of improved materials that have better mechanical and corrosion-protective properties with reduced viscosity and low VOC levels [Jana et al. 2018].

## Alkyd-graphene nanocomposites

### Alkyd-nanofiller composites

**Fig. 4:**
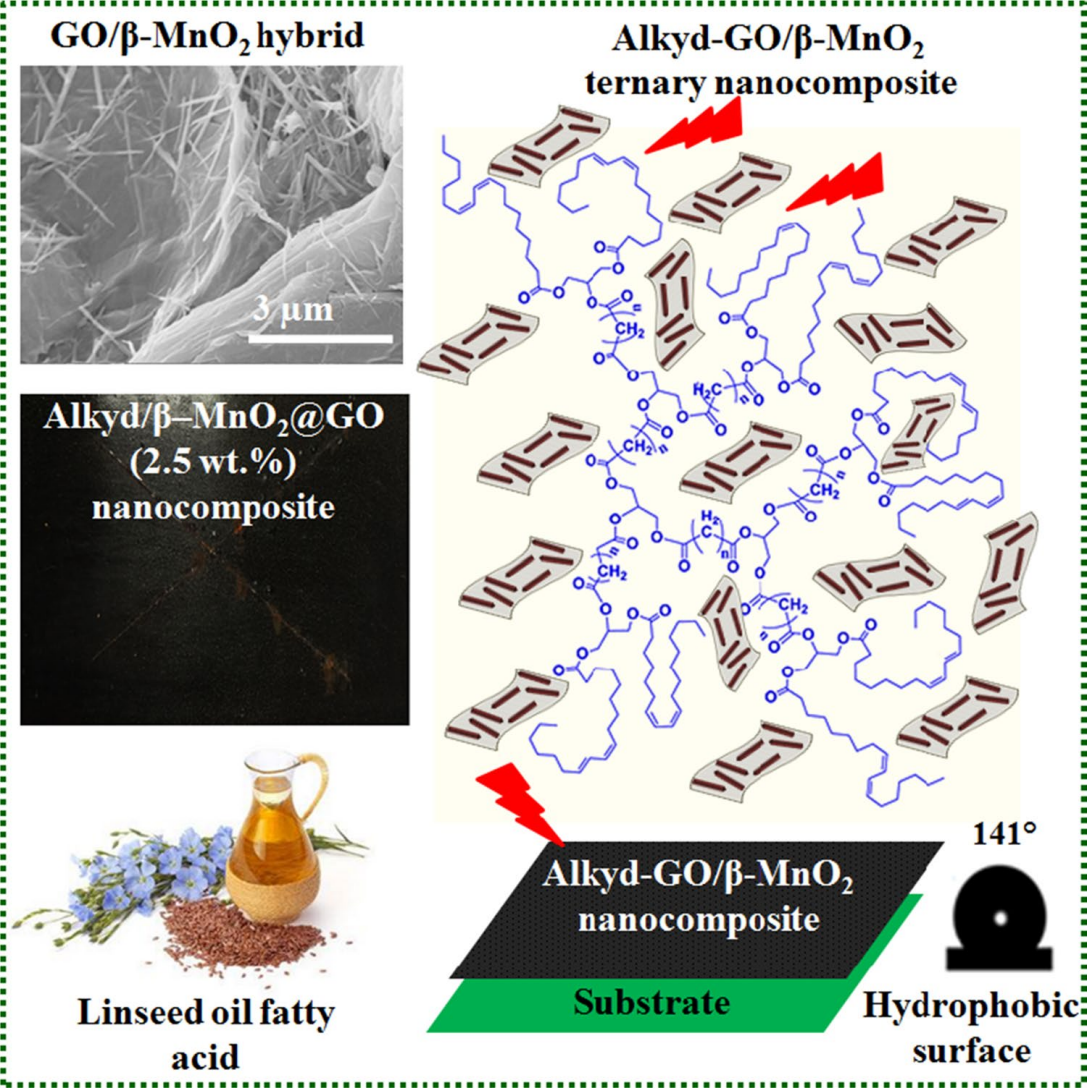
Linseed oil-based hyperbranched alkyds filled with GO/β–MnO_2_ hybrid used as durable and anticorrosion coating materials (Selim et al. 2020a, 2020b). Copyright 2020; reprinted with Elsevier’s permission

**Fig. 5:**
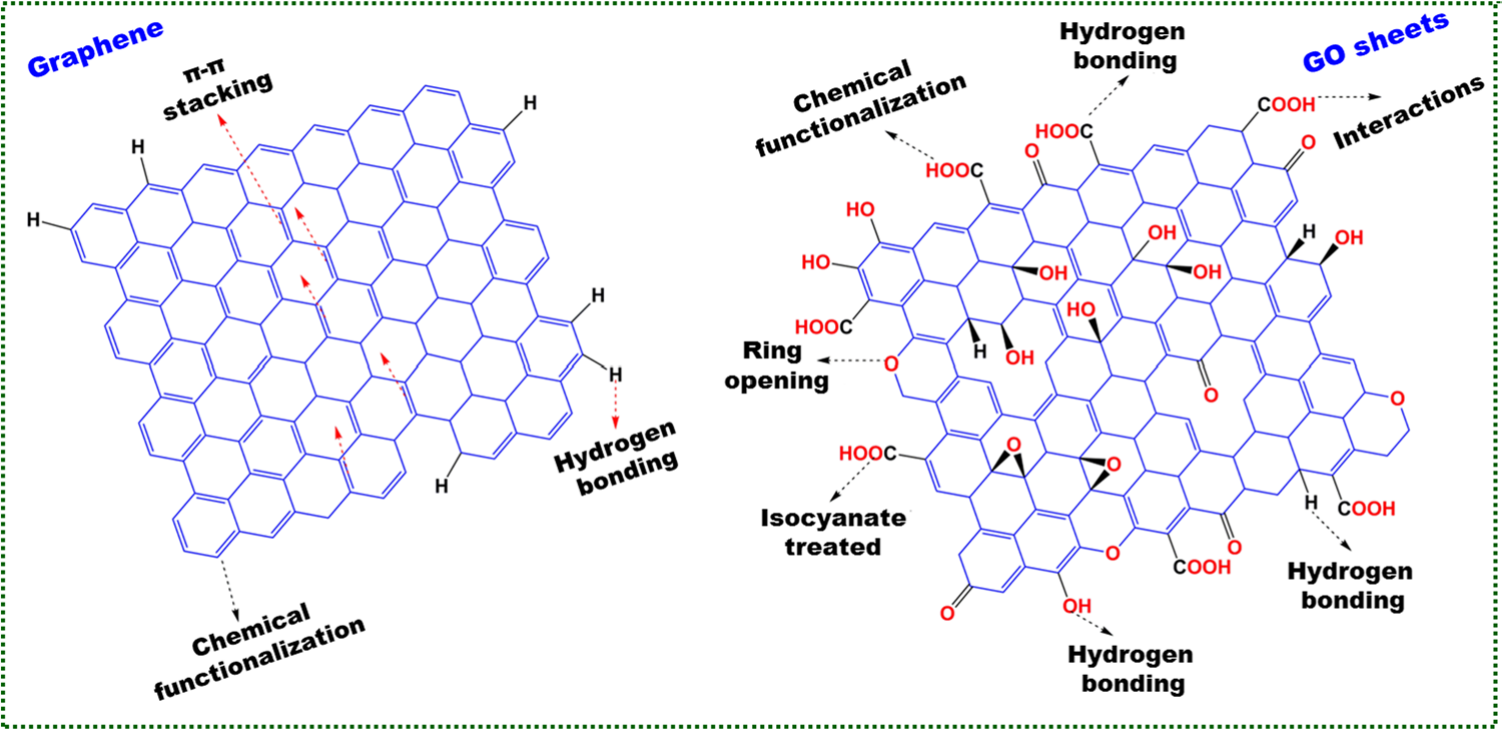
Possible interactions on the surface of **a** graphene and **b** GO materials

The natural occurrence of metallic structural corrosion causes substantial financial and environmental losses [53]. Applying a coated surface is the most efficient way to prevent metallic corrosion and reduce significant corrosion losses [Maya-Visuet et al. 2015]. The alkyd resins have facile preparation methods, ease of application, cost-savings, and are frequently used for corrosion protection [Zhang et al. 2012a, 2012b]. Polymeric alkyds represent 70% of today’s worldwide coating resins [Hlaing and Oo 2008]. International environmental issues linked to using harmful VOCs have sparked recent research to develop eco-friendly and green technologies [Wang et al. 2019]. Low VOC levels, increased solid content, low viscosity, and high functionality were introduced by the hyperbranched alkyd resins that are synthesized in a single step. A ternary composite coating of linseed oil-based hyperbranched alkyd filled with graphene oxide (GO)/β-MnO_2_ nanorods was created using a solution casting method for corrosion protection of steel surfaces. The synthesized nanocomposite coating was dried by auto-oxidation process using lead, manganese, and cobalt octoates (Figs. 4 and 5) [Selim et al. 2020a, 2020b]. The well-distributed alkyd/GO-β-MnO_2_ (2.5 wt%) demonstrated high hydrophobicity (water contact angle of 141°), low surface free energy, micro/nano-roughness, and chemical resistance against 3N NaOH solution.

However, alkyd resins have limitations such as decreased alkaline resistance and permeability against corrosive media [Deyab et al. 2017]. Alkyd/polyurethane copolymers exhibited greater mechanical and physical properties with high resistance to UV light and abrasion due to the presence of urethane linkages [Ling et al. 2014]. Poly(alkyd-urethane) resin can be prepared using alkyd diols derived from vegetable oils mixed with diisocyanates.

The most widely produced vegetable oils in the world are linseed and soybean oils because they are readily available and renewable [Athawale and Pillay 2001]. In the field of coating application, organic/inorganic nanocomposites can introduce enhanced anti-corrosion properties. To enhance the matrix’s anti-corrosion, electrical, and thermal characteristics, multifunctional nanomaterials such as graphene, carbon nanotubes, fullerenes, and graphene sheets were inserted as nanofillers [Nikolic et al. 2016]. Deyab (2015) reported exceptional corrosion protection capabilities of alkyd/carbon nanotube composites after submersion in 3.5% NaCl solution. The high cost of carbon nanotubes (CNTs) prevents their widespread use in industrial businesses, despite the bio-nanocomposites’ remarkable coating properties [Naik et al. 2015]. Therefore, producing polymer composites at low cost with excellent mechanical characteristics may be a possibility. Sheet-like structures of graphene have the best corrosion-barrier properties among all the other shapes [Li et al. 2008]. GO is a variant of graphene that has O surface functional units on top of a layered nanostructure with edges and basal planes [Ammar et al. 2016].

### GO and its oxygen functional groups

The exceptional lamellar structure, mechanical, chemical, and thermal properties of GO sheets is used as anti-corrosive additives for various coatings. Graphene layers have been regulated and functionalized allowing them to be used in a variety of applications. The IUPAC committee has replaced the word “graphite layers” with “graphene,” which was no longer adequate for describing the structure of a single carbon layer because graphite was a 3D stacking structure [Selim et al. 2023]. GO is a two-dimensional monolayer made entirely of carbon atoms. Recent research has looked into a variety of GO fabrication techniques, the most prevalent of which include mechanical and chemical exfoliation, as well as CVD. To get a high yield of graphene single sheets, chemical synthesis of GO from graphite has recently become popular [Shankar et al. 2023]. Graphite oxide is produced by oxidizing graphite with a variety of oxidizing chemicals, including concentrated H_2_SO_4_, HNO_3_, and KMnO_4_. Graphite oxide has higher oxygen content than the sp^2^ hybridized carbon in the basal plane, as well as the carboxyl and carbonyl groups on the sheet edge’s sp^2^ hybridized carbon. The oxygen functionality creation reduces the bond strength between the sheets of graphite oxide and will increase the distance or interlayer gap among the sheets. Graphite oxide is ultrasonicated in distilled H_2_O or a solvent for producing GO nanosheets due to its oxygen-rich activity. The monolayer GO consists of both sp^2^ and sp^3^-hybridized carbon atoms. The sp^3^ hybridized carbon atoms are covalently bonded to oxygen functional groups (oxidized regions), and sp^2^ hybridized regions are un-oxidized. These sp^3^ carbon atoms are usually located above or below sp^2^ carbon atoms [Akarsh et al. 2022]. The ionization of –COOH and –OH units cause the large negative charges of the GO surface acquired after dispersion in water. Water stability of GO colloids is provided via hydrophilicity and electrostatic repulsion. GO is an exotic carbon material with a varieties of oxygen-containing functional groups, including epoxy and hydroxyl on the basal plane as well as carbonyl and carboxylic groups on edges. These oxygen-containing functional groups on the surface of GO can be precisely tuned by simple chemical methods. Such reactive groups are primed for surface modification reactions, resulting in functionalized graphene-based materials. Graphene composite material properties are based on interfacial bonding between graphene and the host matrix (Figs. 4 and 5) [Selim et al. 2023; Shahnaz et al. 2024]. The hydrogen bonding interaction between the polymer and the GO determines the physico-mechanical properties of GO nanocomposites. Polymer-GO compatibility can be considerably improved by grafting and chemical functionality. The polarity of GO nanosheets makes it difficult to disseminate them in a hydrophobic matrix. The oxygenated surface units operate as a catalyst for GO layer aggregation. The GO surface’s organic functional groups can help it work better with hydrophobic polymers. GO can be functionalized with ethyl isocyanate to give dispersed GO in DMF solvent through epoxy ring opening interaction [Potbhare et al. 2022]. The nanocomposite has poor hydrophobic and mechanical properties due to the uneven dispersion of graphenic nanosheets within the polymeric resins. Coating-substrate adhesion and elasticity are minimized due to the poor polymer-graphene interaction forces.

### GO-based alkyd nanocomposites

A large number of GO nanocomposites have been reported as a result of the interaction between the polymer and nanofillers and the massive amount of nano-GO sheets. GO exhibited excellent anti-corrosion properties in polymer coatings [Jena and Philip 2022; Sethulekshmi et al. 2024; Zhou et al. 2022]. An increase in tribological and corrosion protection was reported for polyurethane/GO nanocomposites [Mo et al. 2015]. Improvements in hydrophobicity and anti-corrosion performance were also achieved for the polymethylmethacrylate/graphene nanocomposite [Chang et al. 2014] and epoxy coating [Liu et al. 2016]. These sheets suffered from the closely-packed multilayer structures due to large surface area and van der Waals interactions. This leads to poor exfoliation and dispersion in the alkyd matrix [Stankovich et al. 2007]. GO aggregation can be prevented and well-dispersion can be achieved by decorating the nanosheets with inorganic nanomaterials, particularly ceramic metal oxides [Gutierrez-Gonzalez et al. 2015]. Yu et al. (2015) created a well-dispersed epoxy/GO-Al_2_O_3_ (2.0 wt%) nanocomposite with enhanced anti-corrosion properties and mechanical robustness. GO-Al_2_O_3_ nanohybrid dispersed more effectively than bare GO sheets [Fan et al. 2010]. A pressure-free GO-Al_2_O_3_ nanocomposite with high wear resistance and mechanical durability was introduced [Kim et al. 2014]. The barrier layer and substrate-adhesion strength of the nanocoatings could be strengthened by exfoliation and well-dispersion of the GO-Al_2_O_3_ hybrid. Increased surface hydrophobicity could increase resistance to corrosive aqueous molecules [Yang et al. 2014]. It is possible to create durable alkyd/graphene nanocomposite coating with high chemical resistance for anti-corrosion and mechanical applications.

## Synthesis of alkyd resins

Fatty acids, poly-hydric alcohols, and di-basic acids are frequently used as sources of alkyd, a polyester resin. It has a variety of uses in the coatings manufacturing related to its low-cost, comprehensive competently, and manufacture from sustainable seed-oils. The alkyd refers to specific categories of polyester-resins which have undergone changes brought on by monobasic fatty acids. Their final products are made from seed oil, which also includes monobasic fatty acids, polybasic acid, and polyhydric alcohol (polyol). The word “alkyd” was made by combining alcohol and acid. About midway through the 1920s, Kenley started making alkyd resins [Martens 1961]. After General Electric began large-scale production in 1933, the demand for alkyds as a low-cost and effective paint binder surged. Alkyd resins have received significant interest because of their adaptability and several advantageous properties for a variety of applications [Hofland 2012]. The benefits include a self-oxidation cross-linking process, high brightness, decent color retaining, decent temperature and solvent fight, and good heat resistance. Alkyd resins’ shortcomings include weak resistance to water, acids, and alkalis. Construction and manufacturing coatings are the most common uses for alkyd-based coatings [Wutticharoenwong et al. 2012]. Because the environmental alarms in the 1980s, the developments in alkyd research resulted in the creation of ecologically friendly alkyds. Alkyd suspensions, higher solid-content, and UV-treatable alkyd structures are examples of current alkyd technology, which was developed in reaction to high-emission systems [Wutticharoenwong et al. 2012; Thanamongkollit et al. 2012].

### Common techniques for synthesizing alkyd resin

**Fig. 6:**
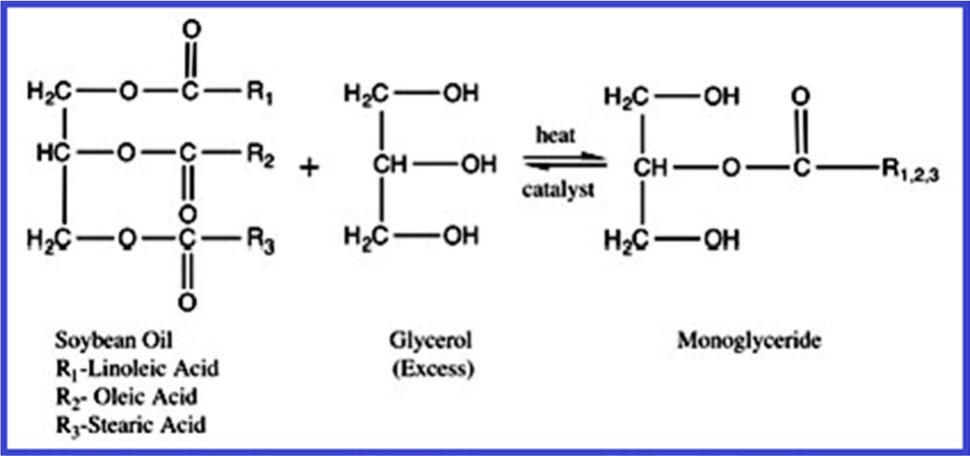
Glycerol and soybean oil are transesterified to create monoglycerides (Soucek and Salata 2021). Copyright 2021, reproduced with permission from Springer Berlin Heidelberg

**Fig. 7:**
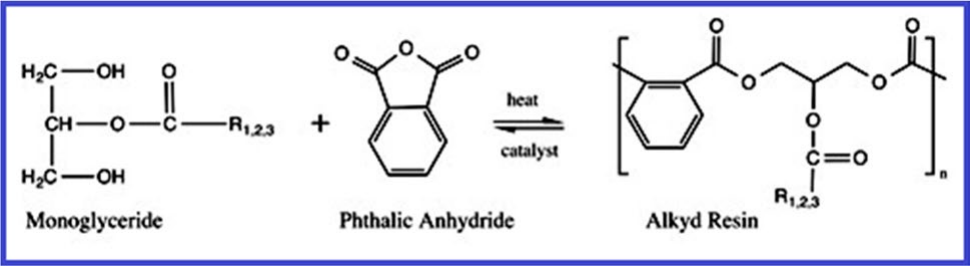
Phthalic anhydride and monoglyceride are polymerized to create alkyd resin (Soucek and Salata 2021). Copyright 2021, reproduced with permission from Springer Berlin Heidelberg

**Fig. 8:**
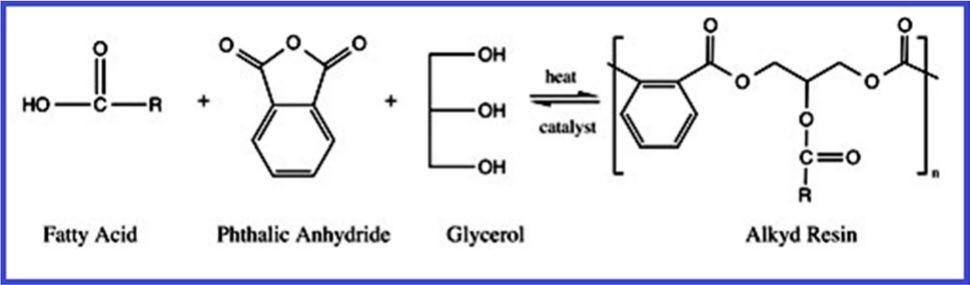
Alkyd resin is polymerized using the fatty acid reaction (Soucek and Salata 2021). Copyright 2021, reproduced with permission from Springer Berlin Heidelberg

**Fig. 9:**
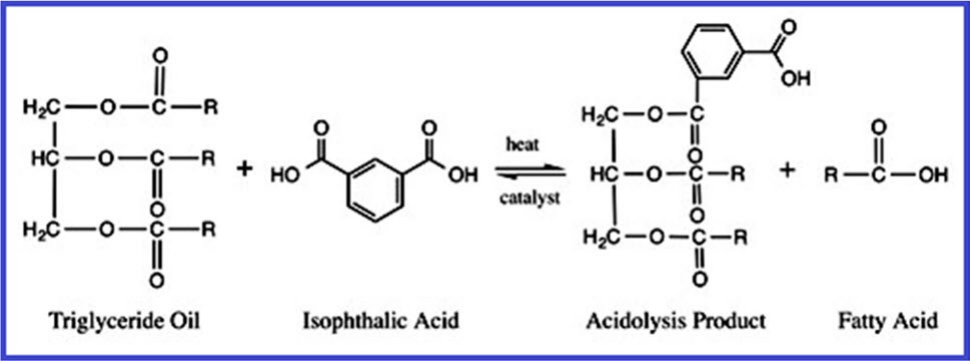
Isophthalic acid is used in an acidolysis reaction to alter triglyceride oil (Soucek and Salata 2021). Copyright 2021, reproduced with permission from Springer Berlin Heidelberg

The mono-glyceride procedure, the fatty-acid procedure, and acidolysis are three typical methods for producing alkyd resins. Glycerol is commonly used as a polyol in the monoglyceride process. Esterification is the first step in this two-stage procedure. The appropriate glycerin content is produced by the oil’s reaction with extra glycerin [Martens 1961; Wutticharoenwong et al. 2012; Thanamongkollit et al. 2012; Wicks Jr. et al. 2006]. Normally, this process is carried out inertly with a catalyst present at temperatures between 230–250 °C. Examples of representative reagents contain Li hydroxide, Li ricinoleate, and tetrapropyl titanate [Soucek and Salata 2021]. The reaction’s byproducts include monoglycerides, diglycerides, unconverted drying oil, and unreacted glycerol. Figure 6 shows an example of glycerol and soybean oil being transesterified [Sailer and Soucek 1999]. The mono-glyceride and a di-basic acid, for example phthalic anhydride, are directly esterified on the second stage, as depicted in Fig. 7. Due to the intricacy of the alkyd polymer, even minor modifications to the reaction environment can have a significant impact on the final products produced [Martens 1961]. The alkyd depends on high instruction polyols, thanks to the one-step fatty acid method. Although it provides superior process control, a drawback is the high-cost of extracting fatty acids by saponifying seed-oils [Thanamongkollit et al. 2012]. The production of fatty acids is depicted in Fig. 8. Triglycerides are modified by the acidolysis process, which substitutes a di-basic acid for one fatty acid. Faster reaction rates are made possible by the acidolysis product’s considerable increase in solubility. The alkyd is created by adding the polyol next [Martens 1961]. Figure 9 depicts how phthalic acid and triglyceride oil react, producing excess stearic acid and an acid breakdown product. Titration analysis of the fatty acid content can be used to gauge how thoroughly the acidolysis has occurred. The extraction of fatty acids requires a lot of time and effort. Phthalic anhydride has a significant propensity to sublimate, making it unsuitable for this procedure [Soucek and Salata 2021].

### Commercial production of alkyd

Alkyd resins can be produced in large numbers using one of two techniques. Either the solvent method or the solventless fusion technique is used for production. These techniques enable producers to produce 100–10,000 gal of alkyd at once [Martens 1961]. Synthesis takes place in a sizable reactor with an inert atmosphere during the fusion process, commonly referred to as a fusion cook. To remove water vapor and unreacted components, the reactor is vented to a fume scrubber after being inundated with inert gas. The finished product is then filtered, packed, and cooled. Although this method is easy to set up and produces many alkyd-resins, it is incompetent and results in significant substantial loss. Reflux is used in the solvent process, also known as the solvent cook, to eliminate water vapor. Water vapor condenses into a separating vessel after mixing with the solvent, evaporating, and condensing. The solvent is recycled after separation, and the amount of water removed can be monitored.

## Process of drying alkyd resins

Alkyds can be divided into three categories: modified versus unmodified, oxidizing versus nonoxidizing, and oil length. Autoxidation is the process by which reacting alkyds, also denoted to as artificial drying-oils, crosslink when oxygen is present [Sailer and Soucek 1999]. Non-oxidizing alkyds are frequently utilized as resins with hydroxyl functions or as plasticizers. These resins need an external cross-linking agent, like urea- or melamine-formaldehyde. Modified alkyds are resins that contain fatty acids, polyols, and polybasic acids in addition to monomers. This is done to enhance particular properties like water compatibility, toughness, and color change resistance. Straight-alkyds offer decent water resistance, good external durability, and mediocre resistance to petroleum solvents. Strong organic acids, concentrated acids, or oxidizing acids can all damage these alkyds. The oil length of an alkyd is determined by the proportion of mono-basic fatty-acids to the overall weight of the polymer. The equation for calculating oil distance is given in the following equation [Soucek et al. 2012].

1 $$Oil\ length=\frac{weight\ of\ oil}{weight\ of\ alkyd- water\ evolved}\times 100$$

There is some disagreement in the area, but in general, alkyds with an oil-distance of in excess of 60 are thought of as long oil alkyds; those with an oil length between 40 and 60 are thought of as medium oil alkyds; and those with an oil length of less than 40 are thought of as short oil alkyds.

## Autoxidation and driers

Autoxidation, a natural process that occurs in the presence of oxygen, causes alkyd resins to cross-link. Heavy metal–based drier can speed up this relatively slow process. During the induction phase, singlet oxygen produces hydroperoxides that break down into free radicals and start the drying process. The hydroperoxides formed during the oxidation are deliberately decomposed by the primary driers. While naturally occurring antioxidants are eaten, unconsumed free radicals begin to form fatty acid double bonds. Peroxy free radicals can be created when this free radical interacts with oxygen. These free radicals combine to produce crosslinking at C–C, R–O–R, and R–O–O–R bond connections through the process of radical-radical reactions. Compounds based on Co, Mg, Fe, Ce, and V are primary driers. The secondary layer coat is dried by salts of Pb, Zr, Bi, Ba, Al, and Sr. Cobalt driers are the most widely used primary driers because of their propensity to modify oxidation-states in reduction-oxidation processes as well as their high reactivity in solvent base and aqueous paints. When cobalt is used solely, several inherent issues arise, including wrinkles and pigment absorption [Soucek et al. 2012; de Boer et al. 2013]. Metal salt combinations and the inclusion of tertiary driers can be used to offset the drawbacks of utilizing only driers. Non-oxidizing metal salts, such as calcium salts, are tertiary or auxiliary driers because they change structural characteristics like hardness, tensile strength, and adsorption reduction. When used in conjunction with primary driers, Ca-salts have slight or no impact on aeration alone but can reduce the overall demand for cobalt and manganese driers and minimize side effects like wrinkles. Before zirconium took its position as a typical addition to encourage secondary drying, lead was frequently used. Because of the probable phase-out of Co catalysts which are genotoxic and carcinogenic, research on alternate drying catalytic systems is widely conducted.

## Waterborne alkyds

The majority of coatings used globally for environmental protection are waterborne coatings, which employ water as the principal solvent [Javadi et al. 2020]. Waterborne coatings are non-flammable and have minimal toxicity, thanks to the reduction or elimination of the usage of VOCs. However, there are several difficulties with aqueous coatings [Wicks Jr. et al. 1993], including (1) poor wettability: wetting agents (i.e., surfactants) are typically included to lower the surface tension of aqueous coatings because water is a poor wetting solvent for the majority of substrates. (2) Pronounced hydrophilicity: water-soluble, water-dispersible, or emulsions with many hydrophilic groups are examples of waterborne coatings. (3) Slow rate of drying: due to the high latent heat of evaporation, waterborne coatings take a long time to dry in the environment. It is important to note that the coatings’ water resistance, chemical stability, corrosion resistance, and mechanical qualities are all negatively impacted by surfactants, hydrophilic groups, and gaps left in the coatings [Sørensen et al. 2009; Butler et al. 2005; Jung et al. 2016]. As a result, part of the long-term corrosion protection properties is sacrificed when solvent-borne coatings are replaced by aqueous coatings. To solve this problem, researchers have changed aqueous alkyd-resins by including nanoscale and microscale metallic and polymeric components into their architectures. Pathan and Ahmad (2013a)] produced butylated-melamine-formaldehyde (BMF) dried aqueous castor alkyd and investigated the alkyd’s anti-corrosion properties using an environmentally friendly solvent (s-triazine ring). The but-oxy clusters and –OH groups of the BMF’s 12-hydroxy-9-cis-octadecenoic acid could create an additional cross-linking reaction that was responsible for the improvement in the anti-corrosion properties. This study demonstrated that adding an s-triazine ring to castor oil–based water-born alkyd resins can provide a high-performance corrosion protection coating material in an environmentally friendly manner [Pathan and Ahmad 2013b]. It was reported that adding nano-Fe_2_O_3_ to aqueous alkyd coatings could improve the coating performance. Even when nano-Fe_2_O_3_ was added at extremely low concentrations (0.05, 0.1, and 0.2%), the corrosion process was delayed. When the loading was raised to 0.3%, an even higher coating performance was attained. Nano-silica has been found to enhance temperature stability, scrape resistance, adhesive characteristics, TGA, and impact fighting of aqueous alkyd resins. ZnO nanoparticles were added by Dhoke et al. (2009) to aqueous alkyd resin, and they found that the abrasion resistance and film compactness had improved. The catalytic effect of ZnO nanoparticles could affect the hardening procedure to form a rigid and intricate network. The mechanical characteristics of the alkyd nanocomposite were significantly enhanced because of the solider connections among the alkyd-matrix and the nano-particles, [Dhoke and Khanna 2019].

## MD simulation, numerical models, and DFT

The efficiency of steel coatings in different acidic media regulates their performance during the coating process [Zhang et al. 2024]. Research on abrasion and protective coatings mainly motivate their wear patterns, chemical and physical criteria, and protective coatings mechanistic within wind/sand climates, and abrasion resistance. The computational methods are used to screen molecular structure by screening the physical movements of molecules and atoms which help in tailoring novel and enhanced functional coatings [Sethi et al. 2020; Moradi and Rezaei 2022]. Numerical simulation is a helpful and vital tool for building accurate models, exploring coating behaviors and abrasions, screening phase transition, as well as heat and mass transport [Guangfeng et al. 2023]. Piao et al. (2010) screened stress spreading in single-layer coatings using the finite element method, where the coating thickness is inversely proportional to the shear stress at the interface. Liu et al. (2014) utilized failure models and Johnson-Cook intrinsic to screen the influence of solid particles shapes by abrasion rates. Lin et al. (2021) screened wind sand particles effects on the failure dynamics of steel structure coating using Ansys software, indicating shear failures near the impact zones, and the risk of annular tears at the contact area. Alghurabi et al. (2021) employed simulation tools to screen the abrasion rates of free-standing screen mesh at lower concentrations (0.5–2%). Zhang et al. (2024) explored the mechanisms and wear patterns of coatings in the face of abrasion using numerical simulation and lab tests. Chen et al. (2023) discussed the mechanism of nitrogen-doped graphene aerogel by an experiment-simulation complementary strategy.

### MD simulation

Experimental methods are unable to depict the molecular scale and material behavior at the interface, so molecular simulation is used to explore the coatings molecular morphologies of liquid materials at the interface (Kizilkaya et al. 2023; Ali et al. 2023). MD simulation is an efficient technique used to manipulate and investigate the complex atomic structure and thermal transport behavior at the nano-scale [Zhao et al. 2020; Nurrohman et al. 2023]. This is based on random sampling and statistical analysis for validating short calculation times for coating processes [Kizilkaya et al. 2023], and a virtual toolbox has been used in the modeling of structure-property and bulk criteria relationship of process optimization and investigate water vapor condensation on various surfaces [Nurrohman et al. 2023; Mostafaei et al. 2023; Souza et al. 2022]. MD simulation is employed in surface coating to model the surface performance, substrate adhesion and bulk properties, comprising surface energies, water/oil contact angles, substrate interaction energy mechanism at the molecular level [Chen et al. 2023], transparency, solubility parameter, and thermal stability [Sethi et al. 2020]. Moreover, it allows the regeneration of a probability distribution function from the given data set [Mostafaei et al. 2023; Souza et al. 2022; Abdulfatai et al. 2019]. MD performed by a dual mesoscopic and coarse-grained molecular dynamics simulation methods through *Ds-Biovia Material Studio* software [El-Hoshoudy et al. 2021a; El-Hoshoudy 2021]. The applied force fields for energy minimization, calculation of atoms interactions, and prediction of structural properties of gas/condensed phase may be “COMPASS” force-field, “steepest descent,” “ABNR,” “quasi-Newton” and “conjugate gradient.”

Structure energy minimization and geometry optimization is performed through “Forcite module” under a constant temperature and pressure system and NVT (constant number, volume, and temperature) using Berendsen and Andersen’s thermostat [Moradi and Rezaei 2022; El-Hoshoudy et al. 2021b]. This can ensure initial structure relaxation and eradicate irrational structure [Chen et al. 2023; El-Hoshoudy et al. 2021b]. The interaction energies are calculated by the Particle-Mesh Ewald method [Chen et al. 2023]. The structure was optimized geometrically using the smart algorithm through quasi-Newton methods, and Newton-Raphson [El-Hoshoudy et al. 2020a, b]. Since the structure is packed in an amorphous supercell of equal dimensions, which transformed into a crystal [Sethi et al. 2020]. The cohesive energy density used to calculate the solubility parameter, and expressed the increase in energy per mole of the matter considering intermolecular forces per unit molar volume are negligible [Moradi and Rezaei 2022; El-Hoshoudy et al. 2020c]. Previous literature discussed the conductance of MD simulation in coating processes. Sethi et al. (2020) employed mutual simulation and experimental tests to screen an optimized coating of poly(vinylacetate) with different polydimethylsiloxane wt% using atomistic and mesoscale simulations. Kizilkaya et al. (2023) screened the molecular morphology of smart polyurethane coatings and amphiphilic dangling chains at the W/O interface using a simulation approach.

### Numerical models

Numerical models can be divided.

#### Turbulence model

This realizable k-ε model is employed for steady-state turbulence calculations as follows [Zhang et al. 2024].

2 $$\frac{\partial }{\partial t}\left(\rho k\right)+\frac{\partial }{\partial xi}\left(\rho {ku}_i\right)=\frac{\partial }{\partial xi}\left[\left(\mu +\frac{\mu_t}{\sigma_k}\right)\frac{\partial }{\partial {x}_i}\right]+{P}_k+{P}_b-{\rho}_{\varepsilon }-{Y}_k$$

3 $$\frac{\partial }{\partial t}\left(\rho \varepsilon \right)+\frac{\partial }{\partial xi}\left(\rho \varepsilon {u}_i\right)=\frac{\partial }{\partial xi}\left[\left(\mu +\frac{\mu_t}{\sigma_{\varepsilon }}\right)\frac{\partial \varepsilon }{\partial {x}_i}\right]+\rho {C}_1{S}_{\varepsilon }-\rho {C}_2\frac{\varepsilon^2}{k+\sqrt{v\varepsilon}}+{S}_{\varepsilon }$$

#### Discrete phase model

It forces the Lagrangian technique to pursue the trajectory of discrete-phase particles, which arise from the differential equations of the forces acting on these particles. These forces formulated in a Cartesian coordinate system [Zhang et al. 2024].

4 $$\frac{du_p}{dt}={F}_D\left(u-{u}_p\right)+\frac{g\left({\rho}_p-\rho \right)}{\rho_p}+F$$

5 $${F}_D=\frac{18\mu }{\rho_p{D}_p^2}\frac{C_D{R}_e}{24}$$

6 $${C}_D={\alpha}_1+\frac{\alpha_2}{\operatorname{Re}}+\frac{\alpha_3}{{\operatorname{Re}}^2}$$

7 $$\operatorname{Re}=\frac{\rho {D}_p}{\mu}\left|u-{u}_p\right|$$

#### Abrasion rate model

The abrasion model is employed to derive the abrasion rate which signifies the material wear extent against a wall. Abrasion is defined as the mass loss per unit of time and area [Zhang et al. 2024]. The abrasion rate is defined as:

$$\operatorname{Re}=\sum \limits_{p=1}^{Np}\frac{\overset{.}{m_pE}}{A_{face}}$$

8 $$E=C(d){v}^nf(a)$$

**Fig. 10:**
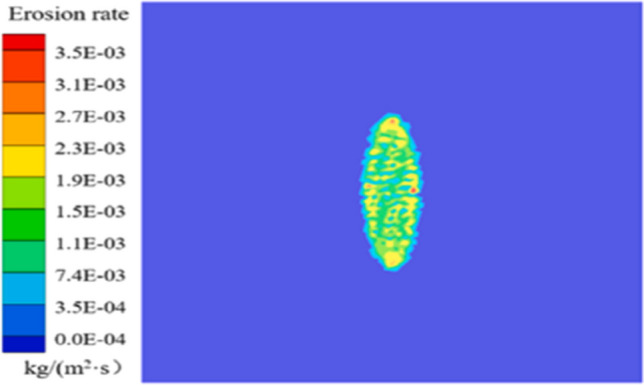
Clouds of erosion rates of graphite/epoxy coatings (Zhang et al. 2024). Copyright 2024, reproduced after the permission from Elsevier

The analysis of graphite/epoxy coatings’ erosion rates through abrasion model simulation can be depicted, as shown in Fig. 10.

#### Three-dimensional model

**Fig. 11:**
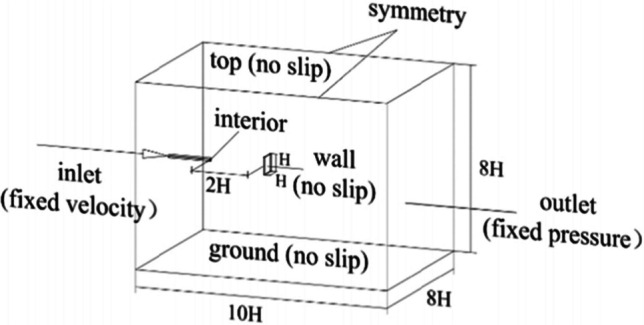
3D model and boundary conditions (Zhang et al. 2024). Copyright 2024, reproduced after the permission from Elsevier

The sandblasting machine’s cabinet is depicted as a quadrilateral basin, while the wear down round pipe diameter range of 10 mm nozzle is as shown in Fig. 11 [Zhang et al. 2024].

### DFT theory

DFT was used to clarify the binding sites related with active radicals during the coating process [Chen et al. 2023]. DFT calculations are employed in the field of corrosion inhibitors and inhibition efficiency [Wang and Qiu 2022]. Additionally, the bonding nature of the interactive moieties can be screened through atom quantum theory [Wang and Qiu 2022]. Kang et al. (2008) calculated graphene adsorption on SiO_2_ surface through quartz (001) crystal using DFT theory with the local density approximation. Li et al. (2022) employ DFT simulation to provide an inclusive assessment of the interactions between graphene and *β*-cristobalite-based SiO_2_ substrates.

## Conclusions

Hyperbranched alkyd nanocomposites represent low environmental hazards. They are used as corrosion-resistant, bacteriophage-resistant, and green coatings. Alkyds could be created at higher temperatures for long reaction times. To reduce the reaction times and temperatures, microwave-assisted techniques for the synthesis of alkyds can be used. More research should be done on waterborne and hyperbranched alkyl nanocomposites. Water-born alkyd nanocomposite would represent a significant scientific advance. This work could review the coated surfaces that are affordable, use natural vegetable oils, and limit pollutants. Alkyd nanocomposites based on vegetable oils represent sustainable alternatives owing to their adaptable structural design, cost reductions, and straightforward manufacturing methods. Graphene materials showed poor exfoliation and dispersion in the alkyd matrixes because of the closely packed layered sheet structures induced by inherent interactions of van der Waals. Polymeric resins’ usage is constrained due to aggregation and inadequate exfoliation. The sheets of GO could be distributed and exfoliated with high compatibility by being decorated with inorganic metals or metal oxides. The graphene hybrid nanofillers can provide excellent dispersion and outstanding anti-corrosion and physico-mechanical coating properties. This shows how even small structural alterations in polymer nanocomposites can drastically improve anti-corrosion characteristics. This work provides a variety of technical advantages for overcoming the drawbacks of metal corrosion which are cost-effectiveness, high-performance, and environmentally friendliness.

## Abbreviations

VOCs: volatile organic compounds
GO: graphene oxide
UV: ultraviolet
MWCNT: multiwall carbon nanotube
BMF: butylated melamine formaldehyde
MD: molecular dynamic
